# Supplementary material for: Commercial fishing amplifies impacts of increasing temperature on predator-prey interactions in marine ecosystems
Source: Nat Commun. 2026 Jan 19;17:628. doi: 10.1038/s41467-025-67362-8 (PMC12815960; doi:10.1038/s41467-025-67362-8)
Supplement: Supplementary file 1 — Supplementary Information [file 41467_2025_67362_MOESM1_ESM.pdf]

Commercial fishing amplifies impacts of increasing temperature on predator-prey interactions in marine ecosystems

Supplementary Information

Amy L. Shurety[1], Murray S. A. Thompson[2], Elena Couce[2], Tom C. Cameron[1] and Eoin J. O'Gorman[1]

[1]*School of Life Sciences, University of Essex, Colchester, UK*

[2]*Centre for Environment, Fisheries and Aquaculture Science, Lowestoft, UK*

Supplementary Figures

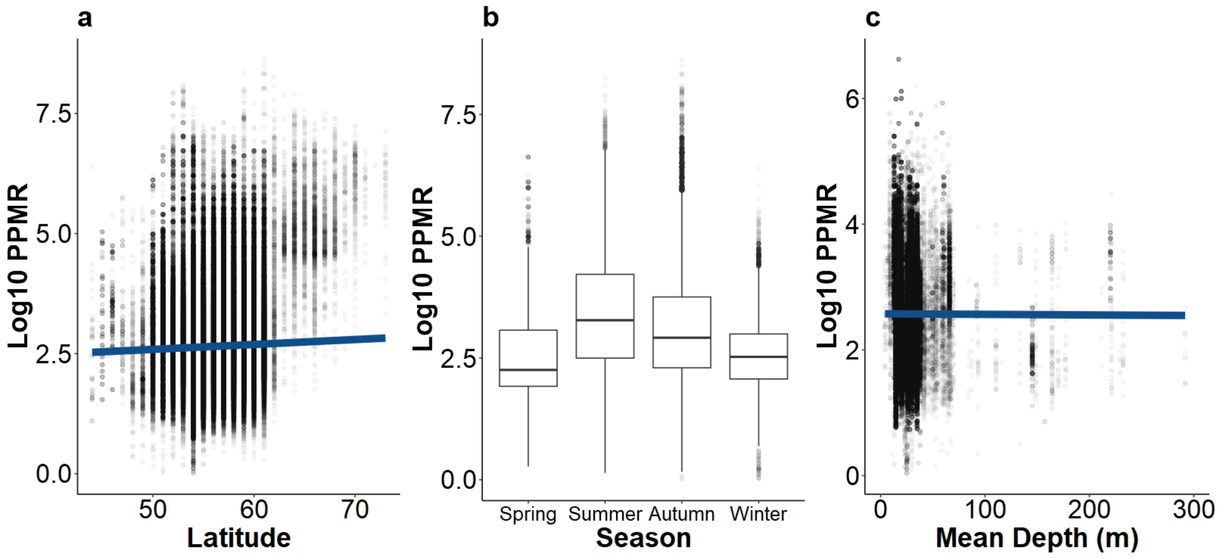

Supplementary Fig. 1: Effects of latitude, season, and mean depth on the predator-prey body mass ratio (PPMR). (a) There was a significant increase in $\log_{10}$ PPMR with increasing latitude (t_{64886} = 27.83, p < 0.001, R^2 = 0.73). (b) There was no significant effect of season on $\log_{10}$ PPMR (F_3= 1.88, p = 0.171). (c) There was no significant effect of depth on log10 PPMR (t_{64886} = 0.92, p = 0.361).

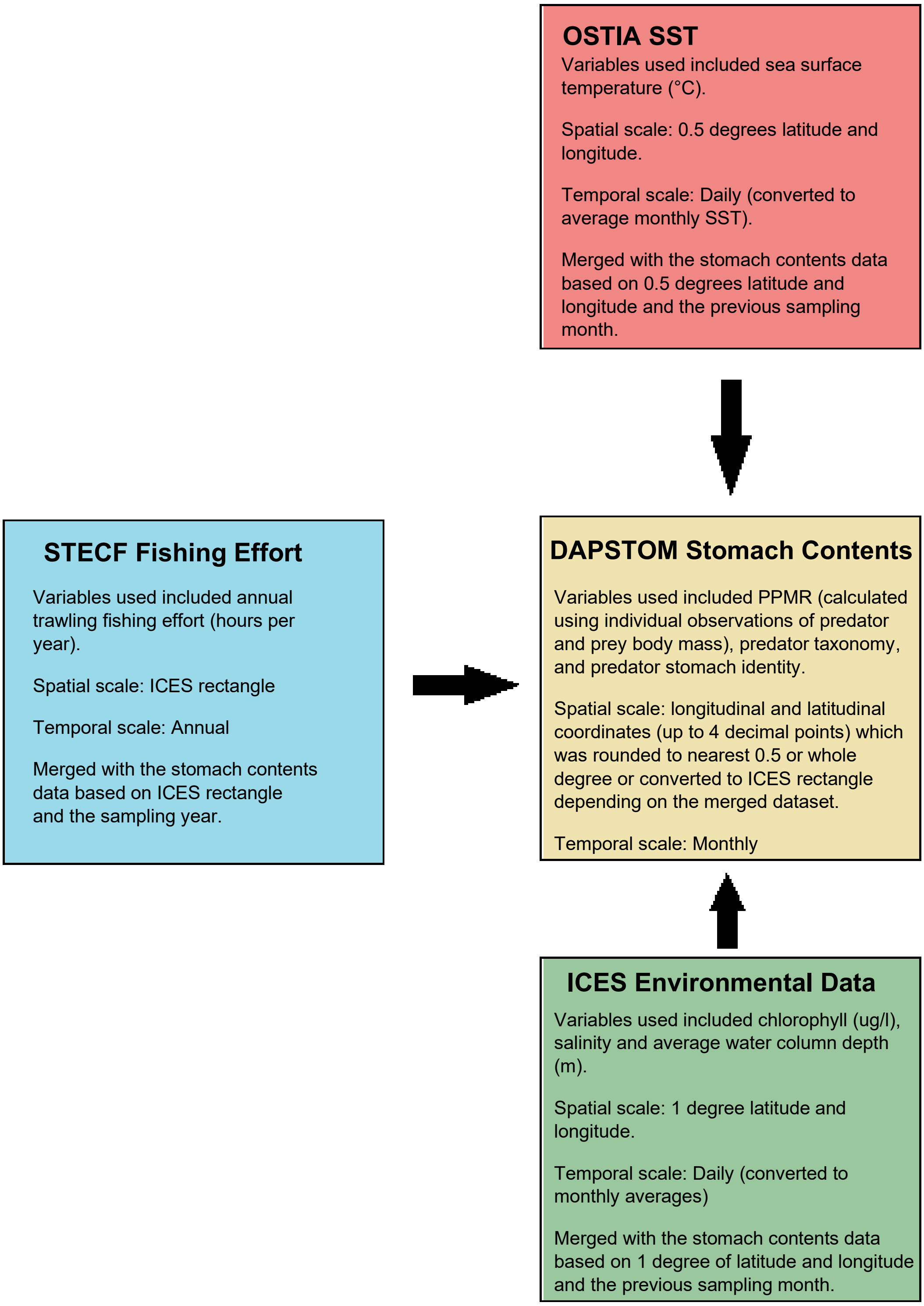

Supplementary Fig. *2*: A flow diagram illustrating the steps taken to join the four different datasets used within this study.

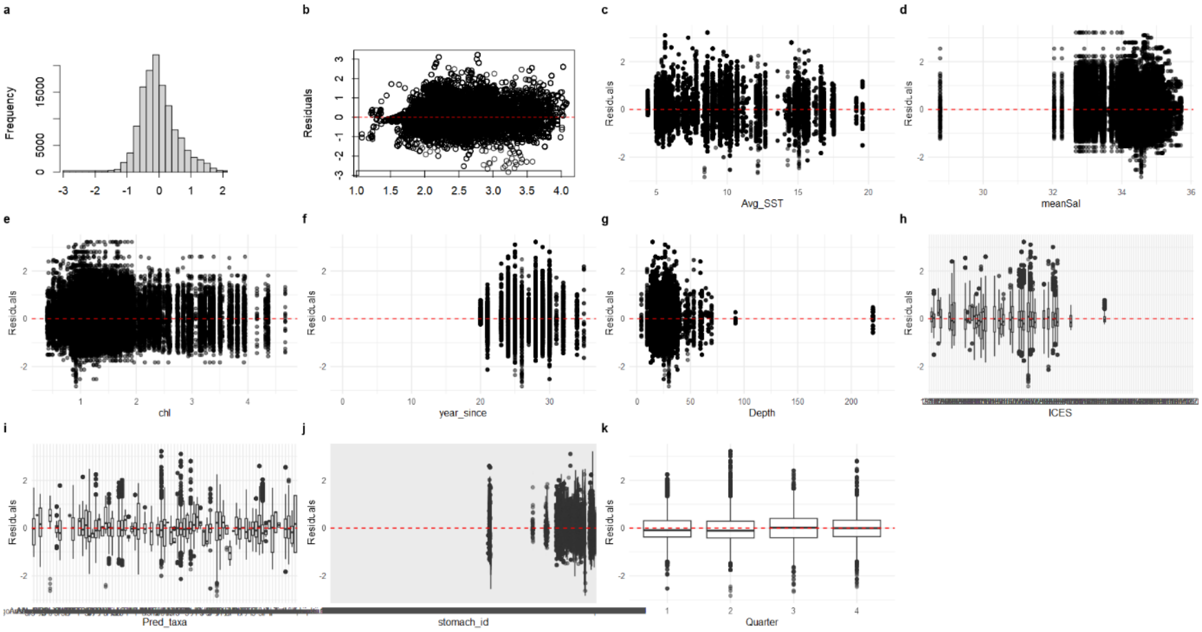

Supplementary Fig. 3: Diagnostic plots of the model for PPMR vs temperature (Figure 1B, n = 64765). (a) Histogram of the residuals, (b) scatterplot of fitted vs residual values, and (c-k) scatterplots of residuals vs each covariate in the model.

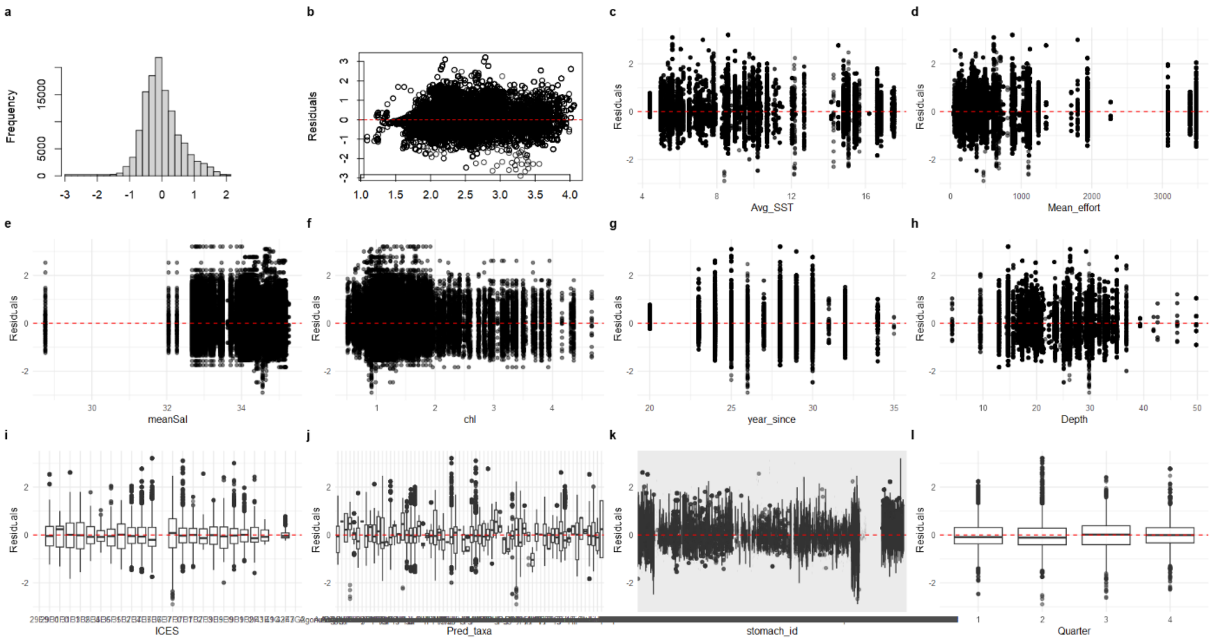

Supplementary Fig. 4: Diagnostic plots of the model for PPMR vs the interaction of temperature and fishing effort (Figure 1C, n = 62437). (a) Histogram of the residuals, (b) Scatterplot of fitted vs residual values, and (c-l) scatterplots of residuals vs each covariate in the model.

Supplementary Tables

Supplementary Table 1: The results of a linear regression of body mass (g) vs temperature (°C) for each individual predator species included in the study with their corresponding percentage of total biomass across all species (%).

Species	**Df**	**slope**	**t-value**	**p-value**	**% biomass**
Agonus cataphractus	954	-0.0122	0.0136	-0.8986	0.0352
Alloteuthis subulata	386	0.0752	0.05	1.5031	0.0131
Amblyraja radiata	3633	0.0028	0.0072	0.3859	0.7332
Ammodytes	14	0.1029	0.2295	0.4486	0.0006
Anarhichas lupus	70	-0.0243	0.0472	-0.5147	0.0327
Anguilla anguilla	30	0.0466	0.0523	0.8925	0.0033
Argentina sphyraena	306	0.0237	0.0705	0.3359	0.0204
Argentinidae	1045	-0.1683	0.0605	-2.7792	0.0708
Arnoglossus laterna	401	0.0217	0.0104	2.079	0.0193
Brosme brosme	10	0.0779	0.1488	0.5235	0.0029
Callionymus lyra	2156	-0.0192	0.0034	-5.7288	0.1416
Capros aper	304	0.0725	0.0758	0.9573	0.0187
Cepola macrophthalma	288	-0.4492	0.1282	-3.5034	0.0215
Chelidonichthys cuculus	3340	0.0253	0.0044	5.7514	0.4646
Chelidonichthys lucerna	968	0.0149	0.0299	0.4994	0.1976
Chelon labrosus	66	-0.0711	0.0785	-0.9061	0.0042
Ciliata mustela	114	-0.0168	0.024	-0.6997	0.0083
Clupea harengus	35106	-0.1035	0.0026	-39.7035	2.7795
Conger conger	334	-0.0734	0.0392	-1.8707	0.1748
Dicentrarchus labrax	127	0.0254	0.03	0.8449	0.0097
Echiichthys vipera	12169	-0.0017	0.0015	-1.0936	0.7304
Enchelyopus cimbrius	248	-0.0206	0.0342	-0.6014	0.0226
Engraulis encrasicolus	26	0.1504	0.1619	0.929	0.0019
Eutrigla gurnardus	39687	0.0114	0.0008	14.1139	4.4904
Gadiculus argenteus	13	-1.0581	3.0759	-0.344	0.0004
Gadus morhua	125677	0.2384	0.0005	488.0396	37.3059
Gaidropsarus vulgaris	260	0.1239	0.0698	1.7758	0.0406
Galeus melastomus	230	-0.1356	0.1159	-1.1692	0.029
Glyptocephalus cynoglossus	254	0.0701	0.062	1.1315	0.0414
Gobiidae	470	-0.0492	0.01	-4.8929	0.0101
Gobiusculus flavescens	129	-0.0233	0.0533	-0.4363	0.0011
Helicolenus dactylopterus	51	-0.4203	0.1921	-2.1884	0.005
Hippoglossoides platessoides	1269	0.0215	0.0085	2.5301	0.1137
Hippoglossus hippoglossus	6	0.1102	0.1573	0.7008	0.0025
Hyperoplus lanceolatus	1148	0.0033	0.005	0.6652	0.0849
Lepidorhombus whiffiagonis	2925	-0.0268	0.0216	-1.2372	0.4381
Leucoraja naevus	543	-0.0525	0.0187	-2.8107	0.1298
Limanda limanda	56388	<0.0001	0.0012	0.0084	5.8963

Lipophrys pholis	224	0.0384	0.0323	1.1902	0.0026
Lophius piscatorius	838	-0.0332	0.0143	-2.3185	0.4105
Maurolicus muelleri	220	0.4669	3.516	0.1328	0.0041
Melanogrammus aeglefinus	59139	-0.0291	0.0009	-31.099	10.1355
Merlangius merlangus	150622	0.1745	0.0004	462.084	14.4708
Merluccius merluccius	2975	0.0851	0.0091	9.3361	0.7765
Microchirus variegatus	264	0.0121	0.0134	0.8987	0.017
Micromesistius poutassou	7800	-0.0204	0.0021	-9.6621	0.594
Microstomus kitt	1507	0.0129	0.0046	2.8082	0.2064
Molva molva	460	-0.0562	0.0374	-1.5021	0.1926
Mullus surmuletus	289	0.0117	0.011	1.066	0.0351
Mustelus asterias	123	-0.4985	0.2059	-2.4216	0.0248
Myoxocephalus scorpius	249	0.0069	0.0144	0.4772	0.0307
Osmerus eperlanus	2	-0.7479	1.2859	-0.5816	<0.0001
Pholis gunnellus	217	-0.1561	0.0189	-8.243	0.0064
Phycis blennoides	2	10.418	11.3788	0.9156	0.0005
Platichthys flesus	847	-0.077	0.0085	-9.0797	0.1326
Pleuronectes platessa	40952	0.0133	0.0013	10.6027	5.3984
Pollachius pollachius	851	-0.0447	0.023	-1.9397	0.41
Pollachius virens	7627	0.0923	0.0031	29.5236	2.7729
Pomatoschistus minutus	13	-0.0082	0.0515	-0.1585	0.0003
Raja clavata	1499	-0.0616	0.0064	-9.5572	0.5425
Raja montagui	646	-0.0422	0.0119	-3.541	0.1523
Sardina pilchardus	56	-0.0077	0.1802	-0.0427	0.0066
Scomber scombrus	27988	-0.0088	0.001	-8.7132	5.3332
Scophthalmus maximus	707	-0.0789	0.0098	-8.0846	0.1912
Scophthalmus rhombus	1781	-0.0799	0.0066	-12.1826	0.5388
Scyliorhinus canicula	3302	-0.0036	0.0073	-0.4983	0.7549
Solea solea	876	0.0442	0.0054	8.207	0.1192
Sprattus sprattus	28697	-0.0155	0.0043	-3.6067	0.9655
Squalus acanthias	965	0.1286	0.01	12.8128	0.3281
Taurulus bubalis	202	-0.2194	0.0168	-13.0942	0.0078
Trachurus trachurus	2875	0.0166	0.0041	4.0323	0.4342
Trigloporus lastoviza	244	-0.004	0.1256	-0.0315	0.0357
Trisopterus esmarkii	577	0.263	0.0104	25.2188	0.0192
Trisopterus luscus	2494	0.0279	0.0037	7.4572	0.3641
Trisopterus minutus	3185	-0.0024	0.0048	-0.5069	0.2222
Zeus faber	1294	0.0369	0.0102	3.6286	0.2667

Supplementary Table 2: The results of a linear regression of body mass (g) vs temperature (°C) for each individual prey species included in the study with their corresponding percentage of total biomass across all species (%).

Species	Df	slope	t-value	p-value	% biomass
Abra alba	4372	-0.0094	0.0052	-1.8208	0.3591
Abra prismatica	26	0.0023	0.0464	0.0489	0.0027
Acartia clausi	67	0.1574	0.0297	5.3038	0.0069
Alloteuthis subulata	70	-0.2914	0.1723	-1.6905	0.0318
Ammodytes	9190	-0.0458	0.0011	-40.3889	4.2664
Ampelisca	710	-0.0403	0.0061	-6.6473	NA
Amphipoda	1248	0.0537	0.0035	15.4342	NA
Amphiura filiformis	6301	-0.1358	0.0019	-72.0354	NA
Anapagurus laevis	29	<0.0001	0.0562	<0.0001	0.0075
Annelida	5532	-0.1012	0.0022	-45.1269	NA
Antalis entalis	44	0.0045	0.0929	0.0487	0.0104
Aphrodita aculeata	675	0.1042	0.0104	10.0331	0.4652
Argentina sphyraena	28	-0.107	0.0268	-3.9961	0.0164
Arnoglossus laterna	28	0.018	0.0242	0.7467	0.0134
Arthropoda	22668	0.2233	0.0015	152.9064	NA
Atelecyclus rotundatus	28	-0.0895	0.0317	-2.827	0.0121
Balanus crenatus	9	-0.04	0.0465	-0.8603	0.0005
Bathyporeia pelagica	28	0.0609	0.1383	0.4404	0.0007
Bodotria scorpioides	12	<0.0001	5.0946	<0.0001	NA
Brachynotus sexdentatus	16	<0.0001	0.1219	<0.0001	0.0052
Buccinum undatum	148	0.2894	0.0559	5.1776	0.0542
Buglossidium luteum	170	-0.0263	0.0101	-2.602	0.1322
Calanoida	2977	-0.0424	0.0053	-7.9871	NA
Calanus	5582	-0.0713	0.0049	-14.6381	NA
Callianassa subterranea	307	<0.0001	0.0357	<0.0001	0.3209
Callionymus lyra	1103	-0.0137	0.0036	-3.7796	0.7601
Calocaris macandreae	1560	-0.0404	0.0069	-5.8901	0.5017
Cancer pagurus	44	-0.1695	0.0274	-6.1905	0.0239
Cancridae	3890	-0.0855	0.0027	-31.6956	1.1772
Candacia armata	55	<0.0001	0.2866	<0.0001	0.0075
Capitella capitata	23	0.1759	0.0514	3.4229	NA
Caprella monocera	27	0.0843	0.0845	0.9979	0.0008
Carcinus maenas	32	0.0381	0.0314	1.2117	0.0373
Cerastoderma edule	13	0.0025	0.0356	0.0707	0.0081
Chlamys opercularis	64	-0.0043	0.0287	-0.151	0.0282
Chordata	13544	0.0107	0.0021	5.0849	NA
Clupea harengus	619	-0.0966	0.0162	-5.9716	0.7648
Clupeidae	226	-0.0178	0.0128	-1.3939	0.1399
Cnidaria	98	-0.1115	0.0157	-7.0942	0.0733
Corophium volutator	20	<0.0001	0.041	<0.0001	0.0005
Corystes cassivelaunus	761	-0.1228	0.0044	-28.0762	0.272
Coscinodiscus	111	0.1815	0.0496	3.6622	0.0119

Crangon allmanni	9	-4.7387	4.0903	-1.1585	0.0024
Crangon crangon	18	-0.1146	0.0412	-2.7835	0.0049
Crangonidae	2624	-0.0276	0.0028	-9.7756	0.724
Crepidula fornicata	16	-0.049	0.049	-0.9992	0.0005
Ctenophora	54	-0.0108	0.0195	-0.5545	0.0129
Cultellus pellucidus	167	-0.1394	0.0134	-10.4412	0.0369
Cumacea	91	0.0171	0.0131	1.3042	NA
Cylichna cylindracea	91	<0.0001	0.1212	<0.0001	0.0157
Decapoda	2873	0.0044	0.0024	1.8256	NA
Dendronotus frondosus	16	4.3066	3.8564	1.1167	0.0018
Diptera	11	-0.0098	0.0486	-0.2022	0.0009
Ebalia cranchii	55	0.1224	0.0288	4.2443	0.0132
Echiichthys vipera	74	-0.0656	0.0127	-5.172	0.0718
Echinocardium cordatum	71	-0.0583	0.0197	-2.9528	0.0357
Echinocyamus pusillus	160	0.0208	0.0102	2.0348	0.011
Echinodermata	132	-0.0129	0.0115	-1.1246	0.0245
Echiurus echiurus	116	-0.0728	0.0181	-4.0284	0.0459
Enchelyopus cimbrius	96	0.6455	0.1125	5.7379	0.1225
Ensis	181	0.0119	0.01	1.1979	0.0553
Eteone longa	65	<0.0001	0.051	<0.0001	0.0021
Euphausiidae	7336	-0.0428	0.0032	-13.5687	1.2991
Euspira	64	<0.0001	0.0322	<0.0001	0.0127
Funiculina quadrangularis	12	<0.0001	0.1698	<0.0001	0.0084
Gadidae	3738	0.6711	0.0088	75.8459	1.6039
Gadus morhua	416	-1.0092	0.2342	-4.3084	0.0389
Gaidropsarus	148	-0.4251	0.0807	-5.2677	0.1372
Galathea	1074	-0.2158	0.0071	-30.5241	0.3446
Galathea intermedia	875	0.007	0.0052	1.3503	0.0729
Gammaridae	158	-0.0242	0.0227	-1.0665	0.0272
Gammarus duebeni	21	<0.0001	0.0412	<0.0001	0.0007
Gari fervensis	28	<0.0001	0.0928	<0.0001	0.0076
Glycera	2607	0.066	0.0055	12.0008	NA
Glyptocephalus cynoglossus	180	-2.4751	0.1586	-15.6076	0.203
Gobiidae	538	-0.0542	0.005	-10.7716	0.1384
Golfingia vulgaris	382	-0.0029	0.0157	-0.1851	0.0922
Goneplax rhomboides	810	-0.0222	0.0154	-1.439	0.5363
Goniada maculata	36	-0.2971	0.0223	-13.3022	0.0068
Gymnammodytes semisquamatus	46	0.2717	0.3118	0.8712	0.0217
Harmothoe impar	26	-0.1224	0.0688	-1.7807	0.0015
Harpacticoida	71	-0.7457	0.0334	-22.3307	0.0029
Hediste diversicolor	137	<0.0001	0.0178	<0.0001	0.0075
Heleobia stagnorum	12	<0.0001	0.0774	<0.0001	NA
Hippoglossoides platessoides	79	0.1032	0.0351	2.9375	0.0836
Hyale nilssoni	24	<0.0001	0.0847	<0.0001	0.0023
Hydroida	204	-0.1477	0.0214	-6.9144	0.0308
Hyperiidae	290	-0.019	0.0049	-3.9091	NA

Hyperoplus lanceolatus	16	0.1773	0.0326	5.4418	0.0149
Idotea	68	0.0457	0.0197	2.326	0.0066
Idotea granulosa	54	<0.0001	0.056	<0.0001	0.001
Inachus leptochirus	39	0.0343	0.0214	1.6063	0.012
Iphimedia	27	-0.0767	0.0286	-2.6768	0.0012
Iphinoe trispinosa	52	-0.5941	0.0656	-9.0504	NA
Isopoda	74	0.0478	0.0469	1.02	0.0053
Lacuna pallidula	15	<0.0001	0.1296	<0.0001	0.0009
Limacina retroversa	15	0.0128	0.3244	0.0395	0.0002
Limanda limanda	371	-0.0599	0.0176	-3.4017	0.3989
Liocarcinus	1288	-0.0744	0.0044	-16.9114	0.5675
Liocarcinus depurator	68	0.1102	0.0206	5.3601	0.0355
Liocarcinus holsatus	47	-0.18	0.02	-9.0211	0.0157
Liocarcinus pusillus	38	-0.1509	0.0192	-7.8766	0.0051
Littorina littorea	21	<0.0001	0.097	<0.0001	0.005
Littorina obtusata	15	<0.0001	0.1296	<0.0001	0.0036
Littorina saxatilis	27	<0.0001	0.0778	<0.0001	0.0038
Loliginidae	378	-0.0683	0.006	-11.3469	0.2244
Lumbrineris	1024	-0.2165	0.0159	-13.5811	0.0877
Macropodia rostrata	256	-0.0836	0.0163	-5.133	0.0802
Mactridae	12	-0.1676	0.0666	-2.5157	0.0065
Majidae	77	-0.3538	0.089	-3.9748	0.0115
Maldanidae	48	1.202	1.9609	0.613	0.0024
Malmgreniella	28	0.3168	0.0779	4.0648	0.0008
Maxmuelleria lankesteri	2173	-0.6442	0.0672	-9.5823	0.9437
Meganyctiphanes norvegica	112	-0.0167	0.0247	-0.6781	0.016
Melanogrammus aeglefinus	348	-0.3033	0.0566	-5.3603	0.6768
Merlangius merlangus	2104	0.0122	0.0145	0.8433	3.195
Microchirus variegatus	176	0.193	0.2457	0.7856	0.2145
Micromesistius poutassou	1572	-0.0661	0.0177	-3.7266	2.5098
Microsetella norvegica	55	-1.2636	0.2866	-4.4095	0.0056
Mollusca	2234	-0.1204	0.0036	-33.8855	NA
Monodonta	12	0.2375	0.3211	0.7395	0.0015
Myctophidae	31	0.0096	0.042	0.2276	0.0091
Mysidae	1035	0.0419	0.004	10.4937	NA
Mysidopsis angusta	2	<0.0001	0.1096	<0.0001	0.0002
Mytilus edulis	5	0.3483	0.0625	5.5742	0.001
Nematoda	123	<0.0001	0.0185	<0.0001	0.0023
Nemertea	303	0.0172	0.0068	2.5441	NA
Nephrops norvegicus	18123	-0.0272	0.0046	-5.9465	14.6755
Nephtys caeca	6976	-0.1712	0.0085	-20.1258	1.8897
Nereis	65	-0.023	0.0119	-1.936	0.015
Nucula	103	-0.109	0.0121	-9.0246	NA
Nudibranchia	12	0.6145	0.9348	0.6573	0.0099
Nyctiphanes couchii	334	-0.0004	0.0187	-0.0194	0.0118
Octopodidae	44	1.0016	0.1365	7.3386	0.1206
Oithona	347	0.1179	0.0103	11.4432	0.018
Ophelina	34	18.3927	3.5009	5.2537	NA

Ophiothrix fragilis	55	0.2875	0.0731	3.9313	0.0225
Ophiura	44	0.0811	0.0199	4.0869	0.0067
Ophiura albida	189	<0.0001	0.0141	<0.0001	0.0401
Ophiura ophiura	90	0.4401	0.0219	20.0589	0.0197
Ophiurida	792	-0.0355	0.0081	-4.3894	0.1882
Paguridae	1294	-0.0509	0.0043	-11.9679	0.4933
Pagurus bernhardus	577	-0.0909	0.0178	-5.1076	0.3267
Palaemon serratus	1117	-0.1793	0.041	-4.3712	0.6051
Palaemonidae	749	-0.066	0.0442	-1.4938	0.4085
Pandalina brevirostris	4	<0.0001	0.1268	<0.0001	0.0006
Pandalus	1974	-0.0199	0.0133	-1.4977	0.6315
Pandalus montagui	1044	-0.0812	0.0104	-7.8233	0.392
Paracalanus	61	0.1308	0.0231	5.6624	0.0021
Parasagitta elegans	134	0.0029	0.0263	0.1115	0.0093
Pasiphaea sivado	1031	-0.005	0.0077	-0.6473	0.3753
Patella vulgata	15	<0.0001	0.1058	<0.0001	0.003
Pectinaria koreni	633	-0.0639	0.0059	-10.849	NA
Pectinidae	101	0.0843	0.0155	5.4325	0.0781
Penaeus	68	<0.0001	0.0616	<0.0001	0.0134
Philine aperta	99	<0.0001	0.0773	<0.0001	0.01
Phoronis muelleri	77	-0.0352	0.0176	-2.0002	NA
Phyllodoce maculata	14	0.0397	0.0961	0.4132	0.0004
Pilumnus hirtellus	87	-0.0249	0.0176	-1.4133	0.0144
Pisidia longicornis	1191	0.0039	0.0032	1.2148	0.0936
Platyhelminthes	16	<0.0001	0.0272	<0.0001	0.0009
Pleuronectes platessa	2934	0.0779	0.0146	5.3288	0.3731
Pleuronectiformes	913	0.0075	0.0133	0.567	0.699
Polinices	15	<0.0001	0.4048	<0.0001	0.001
Pomatoceros triqueter	28	-0.0174	0.0179	-0.969	0.0012
Pomatoschistus minutus	69	0.2127	0.0416	5.1115	0.0156
Porcellana	158	0.0595	0.0379	1.571	0.0273
Processa canaliculata	335	-0.0462	0.0113	-4.0862	0.0466
Psammechinus miliaris	26	-0.5115	0.024	-21.3343	0.0057
Pseudocalanus elongatus	21331	0.1634	0.0086	18.9201	NA
Sabellaria spinulosa	127	0.0123	0.0133	0.9227	0.0073
Sabellidae	35	-0.1701	0.0551	-3.0882	0.0015
Scalibregma inflatum	31	-0.0915	0.0264	-3.4634	0.0022
Scomber scombrus	8	-0.1267	0.0831	-1.5252	0.0058
Semibalanus	93	<0.0001	0.0476	<0.0001	0.0055
Sepiola atlantica	114	-0.0996	0.011	-9.0386	0.0897
Serpulidae	12	<0.0001	0.0916	<0.0001	0.0004
Solea solea	31	0.1283	0.0848	1.5123	0.0256
Soleidae	20	6.2718	0.4231	14.8224	0.0076
Spionidae	22	0.0851	0.0386	2.2046	0.0008
Spisula solida	60	-0.006	0.0257	-0.232	0.0254
Spisula subtruncata	22	<0.0001	0.3167	<0.0001	0.0035
Sprattus sprattus	2378	-0.0109	0.0055	-1.9862	1.5065
Syllidae	38	-0.0153	0.1009	-0.1513	0.0008

Syngnathidae	68	0.041	0.0186	2.204	0.0195
Tellimya ferruginosa	26	<0.0001	0.154	<0.0001	0.0039
Tellina	63	0.0095	0.0213	0.4466	0.012
Temora	14798	0.0967	0.0075	12.8215	NA
Terebellidae	51	-0.1106	0.0292	-3.7897	0.016
Themisto abyssorum	171	-0.0122	0.1341	-0.091	0.0068
Thia scutellata	4	<0.0001	0.2468	<0.0001	0.0015
Timoclea ovata	77	<0.0001	0.0216	<0.0001	0.0118
Trachurus trachurus	12	-0.4853	0.1882	-2.579	0.0068
Trigla	96	0.4594	0.0537	8.5576	0.045
Trisopterus	213	0.1237	0.0528	2.3425	0.09
Trisopterus esmarkii	1470	-0.0028	0.0375	-0.0759	1.5067
Trisopterus minutus	348	-0.0564	0.018	-3.1321	0.3589
Turritella communis	53	-0.0138	0.0752	-0.1838	0.0115
Upogebia deltaura	162	-0.0705	0.0095	-7.4328	0.0833
Varicorbula gibba	50	-0.2932	0.0244	-11.9987	0.005
Xantho pilipes	140	-0.0752	0.1161	-0.6483	0.0991

Supplementary Table 3: The AIC values of all linear and polynomial models tested within this study. The optimal model (AIC value > 2 units lower) is highlighted in bold in each case.

Model	Response variable	Linear	Polynomial
PPMR ~ temperature	PPMR	**254903.3**	255022.9
	Predator mass	**5301.242**	5308.293
	Prey mass	143835.6	**143831.4**
	Prey count	**114131.7**	468990.4
	Prey richness	-85829.94	**-86088.3**
PPMR ~ temperature × fishing effort	PPMR	**246904.1**	246939.1
	Predator mass	**10427.64**	29361.44
	Prey mass	**137925.1**	137996.6
	Prey count	**113464.7**	468703.9
	Prey richness	-80542.45	**-80906.26**
